# Salivary Biomarkers and Their Link to Oncogenic Signaling Pathways in Oral Squamous Cell Carcinoma: Diagnostic and Translational Perspectives in a Narrative Review

**DOI:** 10.32604/or.2025.070871

**Published:** 2025-12-30

**Authors:** Wen-Shou Tan, Hsuan Kuo, Chang-Ge Jiang, Mei-Han Lu, Yi-He Lu, Yung-Li Wang, Ching-Shuen Wang, Thi Thuy Tien Vo, I-Ta Lee

**Affiliations:** 1 School of Dentistry, College of Oral Medicine, Taipei Medical University, Taipei, 11031, Taiwan; 2 Division of Oral and Maxillofacial Surgery, Department of Dentistry, Taipei Medical University Hospital, Taipei, 11031, Taiwan; 3 Faculty of Dentistry, Nguyen Tat Thanh University, Ho Chi Minh City, 700000, Vietnam

**Keywords:** Oral squamous cell carcinoma (OSCC), salivary biomarkers, signaling pathways, non-invasive diagnostics, narrative review

## Abstract

This narrative review examines recent advances in salivary biomarkers for oral squamous cell carcinoma (OSCC), a major subtype of oral cancer with persistently low five-year survival rates due to delayed diagnosis. Saliva has emerged as a noninvasive diagnostic medium capable of reflecting both local tumor activity and systemic physiological changes. Various salivary biomarkers, including microRNAs, cytokines, proteins, metabolites, and exosomes, have been linked to oncogenic signaling pathways involved in tumor progression, immune modulation, and therapeutic resistance. Advances in quantitative polymerase chain reaction, mass spectrometry, and next-generation sequencing have enabled comprehensive biomarker profiling, while point-of-care detection systems and saliva-based omics platforms are accelerating clinical translation. Remaining challenges include variability in salivary composition, lack of standardized collection protocols, and insufficient validation across large patient cohorts. This review highlights the mechanistic relevance, diagnostic potential, and translational challenges of salivary biomarkers in OSCC.

## 1 Introduction

Oral cancer, particularly oral squamous cell carcinoma (OSCC), represents a major global health concern, accounting for approximately 300,000 new cases annually and ranking among the most prevalent malignancies of the head and neck region [1,2]. Despite continuous advances in surgical procedures, radiotherapy, and chemotherapy, a substantial proportion of oral cancer cases, up to 50 percent, are still diagnosed at advanced stages, leading to poor prognosis and reduced survival rates [2]. Conversely, early detection at initial stages (I and II) markedly improves treatment outcomes, with five-year survival rates increasing from approximately 40 percent in advanced disease to about 80 to 90 percent in early-stage OSCC [3,4]. Conventional methods for diagnosing oral cancer, including visual examination, biopsies, and imaging techniques, have important limitations [5]. Visual-tactile examinations rely heavily on the clinician’s experience and may fail to identify early or subtle lesions, resulting in delayed diagnosis. Biopsy procedures, while considered the diagnostic gold standard, are invasive, may cause discomfort for patients, and are not practical for large-scale screening [1]. Consequently, there is growing interest in alternative, non-invasive diagnostic approaches that can complement existing clinical pathways [6].

Saliva has emerged as a promising biofluid for oral cancer detection because it is simple to collect, well-tolerated by patients, and in close proximity to oral lesions, reflecting both local and systemic physiological changes [7]. Saliva contains a diverse range of molecules, including microRNAs (miRNAs), cytokines, proteins, metabolites, and exosomes, many of which are closely associated with molecular mechanisms involved in carcinogenesis, such as alterations in key signaling pathways that regulate cell proliferation, apoptosis, immune responses, angiogenesis, and tumor progression [3,8]. Notably, signaling cascades such as nuclear factor kappa-light-chain-enhancer of activated B cells (NF-κB), mitogen-activated protein kinase (MAPK), phosphoinositide 3-kinase/protein Kinase B (PI3K/Akt), and Janus kinase/signal transducer and activator of transcription (JAK/STAT) play critical roles in inflammation, cell survival, angiogenesis, and metastasis in OSCC [9,10]. Several salivary biomarkers, including specific miRNAs and cytokines, have been shown to influence or reflect the activity of these pathways, offering valuable insights into not only diagnosis but also tumor biology and potential therapeutic targets [11,12]. Over the past decade, significant progress has been made in identifying and analyzing salivary biomarkers for OSCC detection, supported by advances in molecular techniques such as quantitative polymerase chain reaction (qPCR), next-generation sequencing, and mass spectrometry [11,13,14]. However, challenges remain, including variability in salivary composition influenced by individual factors, lifestyle habits, and inconsistencies in sample collection and processing protocols [14]. Moreover, many promising biomarkers still require validation in large and diverse patient populations to enable widespread clinical application [1,7].

This narrative review aims to summarize recent advances in salivary biomarker research for oral cancer detection, with particular emphasis on how these biomarkers relate to signaling pathways implicated in tumor biology. It also highlights analytical technologies, discusses existing challenges, and explores future directions for translating salivary diagnostics into routine clinical practice, noting that saliva-based testing has also been explored in other malignancies such as breast, pancreatic, and lung cancers.

## 2 Salivary Composition and Relevance to Signaling Pathways in Oral Cancer

Saliva is a complex biological fluid primarily secreted by the parotid, submandibular, and sublingual glands, with additional contributions from numerous minor salivary glands distributed throughout the oral mucosa [15]. Saliva contains diverse biomolecules relevant to OSCC detection, such as proteins, nucleic acids, and enzymes [16]. This molecular diversity provides insights into various health conditions, including malignancies such as OSCC [3,6,17]. Several components of saliva have attracted particular interest as potential biomarkers for oral cancer. Dysregulation of these proteins may indicate pathological changes within the oral cavity and can reflect the activation of signaling pathways involved in inflammation and tumor progression [5,9,10,17]. For example, Ferrari et al. demonstrated that elevated levels of specific salivary proteins are associated with increased inflammation and cancer-related tissue changes in OSCC patients [9]. Rai et al. discussed how proteomic analyses of saliva have identified proteins implicated in pathways such as NF-κB and MAPK signaling, which regulate cell proliferation, apoptosis, and immune responses in cancer development [10]. Similarly, Khurshid et al. provided a comprehensive overview of salivary proteomic markers and emphasized their promise not only in detecting OSCC but also in reflecting the biological changes associated with tumor progression and treatment response [17].

Cytokines and growth factors, such as interleukin-6 (IL-6), interleukin-8 (IL-8), and tumor necrosis factor-alpha (TNF-α), are often elevated in the saliva of patients with OSCC, indicating tumor-associated inflammation. These molecules are critical mediators in signaling pathways such as NF-κB, JAK/STAT, and MAPK, which influence cancer cell proliferation, survival, and modulation of the immune microenvironment [9,10].

miRNAs are small, non-coding RNA molecules that regulate gene expression at the post-transcriptional level and remain stable in saliva. Specific salivary miRNAs, including miR-31, miR-125a, and miR-200a, have been linked to OSCC and are under investigation as potential diagnostic tools. Many of these miRNAs directly modulate signaling cascades implicated in oncogenesis, such as PI3K/Akt, transforming growth factor-beta (TGF-β), and Wnt pathways, highlighting their potential role in reflecting tumor biology and therapeutic response [11,12]. Exosomes are extracellular vesicles that facilitate intercellular communication by transporting proteins, lipids, and nucleic acids. Tumor-derived exosomes identified in saliva can carry cancer-specific molecular signatures, including oncogenic miRNAs and mutant DNA fragments, which serve as indicators of disrupted signaling pathways within tumor cells [18].

Metabolites in saliva, such as amino acids, polyamines, and lipids, display altered profiles in patients with OSCC. These metabolic shifts often result from aberrant cellular signaling and metabolic reprogramming typical of cancer cells. Studies have reported significant differences in salivary metabolomic profiles between healthy individuals and OSCC patients, highlighting potential diagnostic applications for these molecular changes [6,13,17,19,20]. Panneerselvam et al. conducted a systematic review demonstrating how metabolomic analyses can distinguish OSCC patients based on specific patterns of metabolites [13], while Zhang et al. emphasized the broader utility of salivary metabolomics in biomarker discovery and disease monitoring [20]. Another review by Khurshid et al. further discussed the potential integration of metabolomic data with other molecular markers for improving diagnostic accuracy in oral cancer [17]. Salivaomics technologies, including proteomics, genomics, transcriptomics, and metabolomics, enable comprehensive profiling of salivary components, while saliva’s non-invasive, low-cost, and easily accessible nature makes it well-suited for large-scale cancer screening and routine disease monitoring [6,14,17,21].

In addition, saliva collection requires minimal training and no specialized equipment, allowing rapid sample processing and integration into standard laboratory workflows, which facilitates its use by clinical laboratories and pathologists. However, the composition of saliva is influenced by factors such as hydration status, circadian rhythms, oral hygiene, smoking, alcohol consumption, and systemic health conditions [13,19]. It is essential that these variables be carefully managed in research and clinical applications to ensure reliable and reproducible biomarker analyses.

## 3 Salivary Biomarkers and Their Association with Signaling Pathways

Early detection of oral cancer significantly improves treatment outcomes and patient survival rates. In recent years, research has increasingly focused on the use of salivary biomarkers as non-invasive and reliable tools for early diagnosis. Among these biomarkers, cytokines, miRNAs, and exosomes have shown particular promise, not only as diagnostic indicators but also as key players in cancer-related signaling pathways. This section explores each biomarker type, highlighting their biological roles, mechanisms, and diagnostic potential in the context of oral cancer.

### 3.1 Cytokines and Related Signaling Pathways

Cytokines in the tumor microenvironment are key regulators of cancer hallmarks, including proliferation, angiogenesis, and metastasis [22]. In OSCC, pro-inflammatory cytokines such as IL-6, IL-8, and TNF-α are consistently elevated in saliva, with reported mean IL-6 concentrations ranging from approximately 30 to 70 pg/mL in patients compared with <10 pg/mL in healthy controls, and IL-8 levels frequently exceeding 200 pg/mL versus baseline values below 50 pg/mL, reflecting active tumor-driven inflammation [9,10,17]. These molecules modulate signaling pathways such as JAK/STAT, NF-κB, and MAPK. For example, IL-6 activates the JAK/STAT3 axis to promote tumor growth and immune evasion [23], while TNF-α and IL-8 enhance tumor invasion via NF-κB and MAPK signaling [24]. Salivary cytokine profiling thus offers insights into both disease activity and underlying oncogenic signaling mechanisms. In addition to their diagnostic role, elevated salivary levels of cytokines such as IL-6 and IL-8 have been associated with recurrence and poor prognosis in OSCC [25]. Furthermore, longitudinal analyses have demonstrated that postoperative trajectories of salivary IL-6 can provide valuable information for disease monitoring [26].

### 3.2 miRNAs and Modulation of Signaling Networks

miRNAs are small non-coding RNA molecules, typically 19–22 nucleotides in length, that regulate gene expression post-transcriptionally by binding to complementary sequences in the 3^′^ untranslated regions (UTRs) of target mRNAs, leading to mRNA degradation or translational repression. In cancer, miRNAs can function as either oncogenes or tumor suppressors, with expression patterns often specific to tissue and tumor type. Salivary miRNAs are highly stable and have emerged as promising non-invasive biomarkers for OSCC. For instance, miR-21 is frequently overexpressed and promotes tumor progression by targeting tumor suppressors and activating the PI3K/Akt and TGF-β signaling pathways [11,27]. Other miRNAs, including miR-31, miR-125a, and miR-200a, have been associated with tumor progression and poor clinical outcomes, partly through modulation of signaling cascades like MAPK and Wnt/β-catenin pathways [11,12]. Recent studies have demonstrated the feasibility of using diagnostic panels comprising multiple miRNAs to achieve high diagnostic accuracy. For instance, one study identified a salivary miRNA panel consisting of eight miRNAs, which achieved an area under the receiver operating characteristic curve (AUC) of 0.954, with a sensitivity of 86% and specificity of 90% for distinguishing OSCC patients from healthy individuals [11]. Technological advances, including quantitative real-time polymerase chain reaction (qRT-PCR) and next-generation sequencing, have enhanced the detection and quantification of salivary miRNAs, enabling their integration into clinical workflows for early diagnosis and monitoring of oral cancer [12]. Certain miRNAs, particularly miR-21, have been more robustly implicated in predicting poor prognosis and therapy resistance [28,29]. In parallel, miR-200a has shown promise in modulating immune response or therapy sensitivity in specific cancer types, suggesting a dual diagnostic-prognostic potential in selected contexts [30–32].

### 3.3 Exosomes as Mediators of Signal Transduction

Exosomes are nanosized extracellular vesicles (30 to 150 nm) secreted by various cell types, including tumor cells, and are present in body fluids such as saliva. They carry a variety of bioactive molecules, including proteins, lipids, mRNAs, miRNAs, and DNA, which reflect the physiological state of their cells of origin [18,33,34]. In cancer, tumor-derived exosomes facilitate intercellular communication by delivering oncogenic cargo that activates key signaling pathways such as NF-κB, PI3K/Akt, and MAPK, promoting tumor growth, invasion, and immune evasion [18,34]. Salivary exosomes serve as a stable and non-invasive diagnostic source because their lipid bilayer protects internal contents from enzymatic degradation [33]. In patients with OSCC, elevated levels of oncogenic miRNAs such as miR-21, miR-24, and miR-200c, as well as proteins including CD63, epidermal growth factor receptor (EGFR), and heat shock protein 70 (HSP70), have been identified in salivary exosomes, distinguishing them from healthy individuals [34]. These features support the diagnostic utility of salivary exosomes and provide advantages over whole saliva in terms of biomarker stability and specificity. Another important feature of exosomes is the presence of exosomal DNA (exoDNA), which can harbor tumor-specific genetic alterations such as TP53 mutations. Detection of such mutations in salivary exosomes offers a non-invasive method for early cancer diagnosis and monitoring minimal residual disease (MRD) during treatment and follow-up. This approach enables clinicians to track tumor-specific genetic changes, assess treatment response, and detect early recurrence, ultimately facilitating personalized patient management [18]. Beyond early detection, salivary exosomal DNA mutations and exosomal miRNA panels are being explored for recurrence monitoring and minimal residual disease assessment, reinforcing their prognostic and predictive potential [35–37]. Several studies have reported that panels of exosomal miRNAs can achieve high diagnostic performance, with sensitivities and specificities exceeding 80 percent in distinguishing OSCC patients from healthy controls [34]. These panels, consisting of miRNAs such as miR-19a, miR-27b, miR-28-3p, and miR-486-5p, reflect underlying oncogenic processes and may serve as complementary tools to traditional diagnostic methods [34].

Despite their promising potential, challenges remain in translating exosomal biomarkers into clinical practice. Significant technical obstacles, such as variability in exosome isolation methods and inconsistencies in analytical techniques, currently limit reproducibility and standardization across studies [18,34]. Furthermore, factors like inflammation, oral infections, and lifestyle habits such as smoking and alcohol consumption can influence the composition and quantity of salivary exosomes, potentially affecting their diagnostic accuracy. The diagnostic performance of exosomes may also be influenced by pre-analytical factors such as saliva collection protocols, processing time, and storage conditions [38]. Rigorous validation in large, multicenter cohorts is essential before salivary exosome-based diagnostics can be routinely implemented in clinical practice. Overall, salivary exosomes hold considerable promise as a non-invasive and highly informative source of biomarkers, with the potential to complement traditional diagnostic methods and enhance early detection, disease monitoring, and personalized therapeutic strategies in OSCC [33,34]. Beyond cytokines, miRNAs, exosomes, and metabolites, other biomarker classes such as DNA methylation signatures and long non-coding RNAs (lncRNAs) have also shown promise in OSCC detection [39]. Aberrant DNA methylation of tumor suppressor genes, including DAPK1, LRPPRC, RAB6C, and ZNF471, has been reported in salivary DNA from OSCC and premalignant lesions, reflecting early epigenetic alterations [40,41]. Similarly, salivary lncRNA MALAT1 was significantly upregulated in OSCC patients compared to healthy controls, achieving 95% sensitivity and 90% specificity in a recent study [42]. Among epigenetic and transcriptional biomarkers, DNA methylation markers such as DAPK1 and ZNF471 have been investigated as potential early detection or tumor suppressor–associated signatures in OSCC, whereas lncRNAs such as MALAT1 and HOTAIR have shown promise as salivary biomarkers that warrant further validation [43–46]. Importantly, several of these biomarkers are currently under clinical investigation (ClinicalTrials.gov Identifiers: NCT05791149; NCT05821179; NCT05708209), underscoring their translational potential. Table 1 summarizes the major salivary biomarker categories in OSCC, including cytokines, miRNAs, exosomes, metabolites, DNA methylation markers, and lncRNAs, along with their representative molecules, associated signaling pathways, and diagnostic relevance.

**Table 1:** Salivary biomarkers, associated signaling pathways, and their clinical relevance in OSCC

Biomarker type	Examples	Associated signaling pathways	Biological role	Clinical relevance (Diagnostic/Prognostic)	References
Cytokines	IL-6, IL-8, TNF-α	NF-κB, JAK/STAT, MAPK	Inflammation, immune modulation, tumor growth	Diagnostic and prognostic-Elevated IL-6 and IL-8 levels correlate with recurrence and poor prognosis; IL-6 trajectories are useful for postoperative monitoring	[9,17,23,26]
miRNAs	miR-21, miR-125a, miR-31, miR-200a	PI3K/Akt, TGF-β, Wnt/β-catenin, MAPK	Regulate apoptosis, proliferation, metastasis	Diagnostic and predictive miR-21 promotes therapy resistance and poor prognosis; miR-200a is associated with immune modulation and therapy response	[27–29,32]
Exosomes	miR-21, CD63, HSP70, TP53 mutations	NF-κB, MAPK, PI3K/Akt	Intercellular signaling, immune evasion, metastasis	Diagnostic, prognostic, and MRD monitoring-Exosomal miRNAs and TP53 mutations enable recurrence tracking and minimal residual disease detection	[18,33,34,36]
Metabolites	Polyamines, amino acids, lipids	Metabolic reprogramming	Reflect biochemical shifts and altered energy metabolism	Diagnostic-Differentiate OSCC from controls using metabolic fingerprints	[6,13,17,19,20]
DNA methylation	DAPK1, LRPPRC, RAB6C, ZNF471	Epigenetic regulation of tumor suppressor pathways	Reflect early epigenetic alterations and gene silencing	Diagnostic-Used for early detection and risk stratification in premalignant and malignant lesions	[40,41]
lncRNAs	MALAT1, HOTAIR	PI3K/Akt, TGF-β/SMAD	Regulate transcription, EMT, and oncogenic signaling	Diagnostic and prognostic-MALAT1 is elevated in OSCC saliva and linked with lymph node status and disease severity	[42–45]

Note: IL, interleukin; TNF-α, tumor necrosis factor-alpha; NF-κB, nuclear factor kappa-light-chain-enhancer of activated B cells; JAK/STAT, Janus kinase/signal transducer and activator of transcription; MAPK, mitogen-activated protein kinase; PI3K/Akt, phosphoinositide 3-kinase/protein kinase B; TGF-β, transforming growth factor-beta; Wnt/β-catenin, wingless-related integration site/beta-catenin; CD63, cluster of differentiation 63; HSP70, heat shock protein 70; TP53, tumor protein p53; MRD, minimal residual disease; DAPK1, death-associated protein kinase 1; LRPPRC, leucine-rich pentatricopeptide repeat-containing protein; RAB6C, Ras-related protein Rab-6C; ZNF471, zinc finger protein 471; lncRNA, long non-coding RNA; MALAT1, metastasis-associated lung adenocarcinoma transcript 1; HOTAIR, HOX transcript antisense intergenic RNA; EMT, epithelial–mesenchymal transition; OSCC, oral squamous cell carcinoma.

## 4 Technologies for Salivary Biomarker Detection

Recent advances in analytical technologies have significantly enhanced the sensitivity, specificity, and clinical utility of salivary biomarker detection for OSCC. These innovations have facilitated the identification of molecular alterations related to cancer-associated signaling pathways, thereby promoting the integration of salivary diagnostics into the framework of precision oncology. Conventional approaches such as enzyme-linked immunosorbent assay (ELISA) and RT-PCR continue to serve as reliable methods for detecting proteins and nucleic acids. These are now complemented by advanced techniques that offer higher throughput and multiplexing capacity. qRT-PCR, in particular, has become a cornerstone for analyzing salivary mRNAs and microRNAs. It has validated several OSCC-associated miRNAs, such as miR-31 and miR-125a, which are implicated in MAPK and PI3K/Akt signaling pathways [14]. Next-generation sequencing (NGS) has further expanded diagnostic capabilities by enabling high-throughput transcriptomic profiling of salivary components. This approach facilitates the identification of novel non-coding RNAs and tumor-specific mutations, including alterations in TP53 and PIK3CA. These genetic changes play essential roles in OSCC pathogenesis through disruption of EGFR/PI3K/Akt, JAK/STAT, and TGF-β/SMAD signaling axes [47,48]. In the proteomic domain, liquid chromatography-tandem mass spectrometry (LC-MS/MS) provides detailed profiling of salivary proteins and peptides. Elevated levels of proteins such as CD44 and HSP70 have been identified in OSCC patients and are closely associated with tumor invasion and stress responses mediated by the NF-κB and MAPK signaling pathways [10]. These findings illustrate the dual utility of salivary proteomics for both diagnostic evaluation and elucidation of mechanistic pathways in tumor biology. Emerging nanotechnology-based approaches, including surface-enhanced Raman spectroscopy (SERS), offer ultrasensitive detection of salivary biomarkers. For instance, SERS assays have successfully identified S100P mRNA, a gene linked to calcium signaling and metastasis, indicating its potential utility in non-invasive, real-time OSCC screening [14]. In addition, microfluidic and lab-on-a-chip platforms are under development to enable point-of-care (POC) testing. These compact diagnostic systems allow rapid, multiplexed detection of nucleic acids and proteins, thereby facilitating timely clinical decision-making in both hospital and remote settings [14]. For example, microfluidic-based devices have shown considerable potential for integrating RNA and protein assays in body fluids, which may support early cancer detection, including applications in OSCC [49].

Table 2 presents an overview of major technologies used for detecting salivary biomarkers in OSCC, highlighting their target molecules, clinical applications, advantages, and limitations. Despite these technological advances, several challenges remain. Standardization of pre-analytical procedures, including saliva collection methods, processing protocols, and storage conditions, is critical to ensure data reproducibility and cross-study comparability. Moreover, large-scale clinical validation across diverse populations is necessary to determine diagnostic thresholds and account for confounding variables such as inflammation, smoking, and oral infections [38,48,50]. Collectively, these evolving diagnostic platforms offer a promising foundation for transforming salivary biomarker detection from an experimental method to a clinically actionable tool. By capturing real-time molecular signals reflective of tumor biology and signal transduction, these technologies support earlier diagnosis, personalized treatment, and improved outcomes in OSCC. Moreover, the successful clinical implementation of saliva-based diagnostics, such as HIV antibody testing and SARS-CoV-2 rapid antigen detection, demonstrates the practicality, regulatory acceptance, and scalability of saliva as a diagnostic medium. These established platforms provide important translational benchmarks for developing and validating salivary biomarker assays in OSCC [51,52].

**Table 2:** Technologies for detecting salivary biomarkers in OSCC and their clinical applications

Detection Technology	Target Molecules	Representative Applications/Examples	Advantages	Limitations	References
ELISA	Cytokines (IL-6, IL-8, TNF-α), proteins (CD44, HSP70)	Quantification of inflammatory and stress-related proteins in OSCC saliva; validation of cytokine biomarker levels	Widely available; quantitative; high specificity	Single-analyte detection; limited multiplexing	[9,10,17]
qRT-PCR	mRNAs, miRNAs (miR-21, miR-31, miR-125a, miR-200a)	Quantitative detection of gene expression and miRNA dysregulation linked to PI3K/Akt and MAPK signaling	High sensitivity and specificity; quantitative; clinically established	Requires RNA extraction; affected by RNA degradation	[11,12,14]
NGS	miRNAs, lncRNAs, DNA mutations (TP53, PIK3CA)	High-throughput transcriptomic profiling; discovery of novel non-coding RNAs and tumor-specific mutations	Genome-wide coverage enables discovery	High cost; complex data analysis	[14,47]
LC-MS/MS	Proteins, peptides (CD44, HSP70, S100P)	Comprehensive proteomic analysis; identification of invasion- and stress-associated proteins	High precision; detects multiple analytes simultaneously	Requires specialized equipment; expensive	[10,14]
SERS	mRNAs, metabolites (S100P mRNA)	Ultrasensitive molecular detection for non-invasive OSCC screening	Rapid and highly sensitive; label-free	Requires nanostructured substrates; technical complexity	[14,50]
Microfluidic and lab-on-a-chip platforms	Combined RNA, protein, and metabolite assays	Point-of-care saliva testing for multiplex biomarker analysis	Miniaturized, fast, low sample volume	Still under validation; standardization needed	[14,49]
Nanotechnology-based biosensors	Cytokines, miRNAs, cfDNA	Integration of nanomaterials to improve sensitivity for salivary biomarkers	Enhanced sensitivity; portable for real-time use	Requires calibration and reproducibility validation	[38,50]

Note: ELISA, enzyme-linked immunosorbent assay; qRT-PCR, quantitative reverse transcription polymerase chain reaction; NGS, next-generation sequencing; LC-MS/MS, liquid chromatography-tandem mass spectrometry; SERS, surface-enhanced Raman spectroscopy; RNA, ribonucleic acid; miRNA, microRNA; lncRNA, long non-coding RNA; cfDNA, cell-free DNA; PI3K, phosphoinositide 3-kinase; Akt, protein kinase B; MAPK, mitogen-activated protein kinase; CD44, cluster of differentiation 44; HSP70, heat shock protein 70; S100P, S100 calcium-binding protein P; TP53, tumor protein p53; PIK3CA, phosphatidylinositol-4,5-bisphosphate 3-kinase catalytic subunit alpha; OSCC, oral squamous cell carcinoma.

## 5 Challenges and Limitations in Translating Salivary Biomarkers into Clinical Practice

Salivary biomarkers show strong potential for early detection and longitudinal monitoring of OSCC. However, saliva itself contains a vast amount of biological information, encompassing genomics, transcriptomics, proteomics, metabolomics, microbiome, and microRNAs. This molecular complexity increases the difficulty of identifying disease-specific signals, as non-cancer-related variations may obscure or confound OSCC-specific biomarker patterns. Consequently, several interrelated challenges continue to delay their clinical translation. These limitations involve issues related to pre-analytical variability, biological heterogeneity, technological constraints, limited validation, and regulatory or ethical considerations. One of the primary limitations is the lack of standardized pre-analytical procedures. Differences in collection methods, such as stimulated versus unstimulated saliva, as well as fasting status, time of day, oral hygiene conditions, transport temperatures, centrifugation parameters, and storage durations, can significantly influence the integrity of nucleic acids, stability of proteins, exosome recovery, and metabolic profiles. These inconsistencies reduce reproducibility and hinder comparisons across studies [14,50,53]. Furthermore, the lack of harmonized normalization strategies, including total protein adjustment, internal spike-in controls, and exosome particle counts, complicates benchmarking across laboratories [54]. Biological variability further complicates salivary biomarker interpretation. Physiological environments, such as hormonal status, systemic diseases, and aging, can markedly alter salivary composition and lead to variability in biomarker profiles. Individual differences such as age, sex, ethnicity, dietary habits, circadian rhythm, systemic inflammation, oral microbiota composition, and exposure to tobacco or alcohol, as well as coexisting oral conditions like periodontitis or mucosal inflammation, may all influence salivary cytokines, microRNAs, exosomal contents, and metabolomic signatures. These factors can obscure OSCC-specific alterations and introduce significant analytical noise [15,20].

Technological progress has enabled highly sensitive biomarker detection through platforms such as quantitative real-time polymerase chain reaction, next-generation sequencing, mass spectrometry, nanoparticle-based assays, and microfluidic devices. While these tools provide enhanced analytical depth and precision, their implementation requires advanced instrumentation, trained personnel, and strict quality control measures [1,14]. Additionally, high per-sample costs and extended processing times limit their scalability for routine clinical application, particularly in under-resourced healthcare settings. Analytical variability introduced by batch effects, platform-specific performance, and inconsistent bioinformatics workflows also contributes to reduced reproducibility [54]. From the perspective of clinical validation, most salivary biomarker studies rely on small, single-center, and cross-sectional cohorts. These studies frequently lack ethnic and geographic diversity. Few have evaluated predictive performance for clinical endpoints such as recurrence, minimal residual disease, or treatment response through prospective longitudinal sampling [15]. In addition, biomarker expression may vary across disease stages, particularly during the transition from oral potentially malignant disorders (OPMDs) to OSCC. These stage-dependent changes highlight the need for longitudinal studies to monitor biomarker dynamics and to establish clinically relevant thresholds for early detection and progression risk assessment. Moreover, although many salivary biomarkers have been reported, their individual sensitivity and specificity remain insufficient for reliable clinical application.

Several studies indicate that single biomarkers are rarely adequate to distinguish OSCC from normal or benign conditions, and biomarker panels or combinations have demonstrated superior diagnostic accuracy and robustness [1,7,9]. In addition, external validation, direct comparison with established diagnostic or prognostic tools, and integration into multivariate models remain limited, which delays inclusion in clinical guidelines [14]. Ethical and regulatory concerns add further complexity. Issues involving patient consent, privacy of salivary genomic data, use of biobank resources, and the interpretation of incidental findings must be addressed. These challenges are particularly relevant when considering the risks of false positives, which may lead to unnecessary biopsies or patient anxiety, and false negatives, which may delay timely treatment. Both scenarios raise concerns about the cost-effectiveness of salivary diagnostics at the population level [54]. Table 3 summarizes the key limitations in translating salivary biomarkers into clinical application, including pre-analytical variability, biological heterogeneity, technological and validation gaps, and ethical considerations. Mechanistically, although many salivary studies have identified differential expression of biomarkers, they often lack direct evidence linking these molecules to specific oncogenic signaling pathways within the tumor microenvironment. These pathways include NF-κB, JAK/STAT, PI3K/Akt, MAPK, TGF-β/SMAD, and Wnt/β-catenin. Without functional validation using tools such as pathway-specific reporter assays, clustered regularly interspaced short palindromic repeats (CRISPR)-based gene editing platforms, or phosphoproteomic profiling through liquid chromatography-tandem mass spectrometry (LC-MS/MS), it is difficult to determine whether salivary alterations represent causative biological events or are merely correlative [55]. To overcome these limitations, several strategies should be pursued. These include establishing standardized operating procedures for saliva collection and processing, implementing robust normalization techniques, integrating multi-omic approaches to improve signal clarity, and conducting multicenter prospective clinical trials that stratify results by demographic and clinical variables. It is also essential to harmonize bioinformatics pipelines, perform cost-effectiveness analyses, and validate the mechanistic roles of salivary biomarkers in tumor signaling pathways. Moving from exploratory research to clinical application will require close collaboration among oral medicine specialists, oncologists, molecular biologists, biomedical engineers, data scientists, and regulatory agencies.

**Table 3:** Key challenges and limitations in translating salivary biomarkers into clinical applications

Category	Specific issues	Impact on clinical translation	References
Pre-analytical variability	Differences in saliva collection (stimulated vs. unstimulated), fasting status, time of day, oral hygiene, temperature, centrifugation, and storage duration	Affects stability of nucleic acids, proteins, metabolites, and exosomes; reduces reproducibility across studies	[14,50,53]
Normalization and analytical variability	Lack of standardized normalization methods (e.g., total protein correction, spike-in controls, exosome particle quantification); platform-dependent batch effects	Limits comparability across laboratories and introduces inter-assay variability	[14,54]
Biological heterogeneity	Variations due to age, sex, ethnicity, lifestyle (smoking, alcohol), oral hygiene, systemic inflammation, circadian rhythm, and comorbid oral diseases (e.g., periodontitis)	Confounds OSCC-specific biomarker patterns and increases background noise	[15,20]
Technological and cost barriers	Advanced instruments (qRT-PCR, NGS, LC-MS/MS, microfluidic, nanoparticle-based assays) require expertise, quality control, and are costly	Limits scalability in low-resource settings; restricts population-level screening	[1,14]
Limited validation and clinical evidence	Most studies are single-center, small cohort, cross-sectional; lack longitudinal or multicenter validation; limited data on recurrence, MRD, or therapy response	Hinders establishment of clinical thresholds, reduces generalizability, delays clinical adoption	[15,35–37]
Mechanistic uncertainty	Lack of direct evidence linking salivary biomarker changes to specific tumor signaling pathways (NF-κB, JAK/STAT, PI3K/Akt, MAPK, TGF-β/SMAD, Wnt/β-catenin)	Unclear whether biomarkers are causative or correlative; weakens translational interpretation	[9,10,47,55]
Ethical and regulatory considerations	Patient consent, genomic data privacy, biobank governance, risk of false positives/negatives	Raises concerns about overdiagnosis, patient anxiety, and cost-effectiveness	[53,54]

Note: qRT-PCR, quantitative reverse transcription polymerase chain reaction; NGS, next-generation sequencing; LC-MS/MS, liquid chromatography-tandem mass spectrometry; MRD, minimal residual disease; NF-κB, nuclear factor kappa-light-chain-enhancer of activated B cells; JAK/STAT, Janus kinase/signal transducer and activator of transcription; PI3K/Akt, phosphoinositide 3-kinase/protein kinase B; MAPK, mitogen-activated protein kinase; TGF-β/SMAD, transforming growth factor-beta/mothers against decapentaplegic homolog; Wnt/β-catenin, wingless-related integration site/beta-catenin; OSCC, oral squamous cell carcinoma.

## 6 Future Perspectives on Integrating Salivary Biomarkers and Signaling Pathway Analysis

### 6.1 Integration with Signaling Pathways and Emerging Technologies

The future of salivary biomarkers in OSCC lies not only in their diagnostic utility but also in their capacity to offer mechanistic insights into tumorigenesis through integration with cancer-associated signaling pathways. Emerging evidence suggests that comprehensive, pathway-informed biomarker strategies will be central to advancing precision oral oncology [1,7,9]. A key direction involves the development of multi-omic biomarker panels that combine genomic, epigenomic, transcriptomic, proteomic, and metabolomic data. This systems-level approach enhances diagnostic sensitivity and specificity by capturing the complexity of tumor biology. For example, panels integrating salivary microRNAs with protein biomarkers such as CD44 or HSP70 may better reflect activation of key oncogenic pathways, including NF-κB, JAK/STAT, MAPK, and PI3K/Akt [1,14]. To enable widespread clinical application, future efforts must prioritize the standardization of saliva collection, stabilization, and processing protocols. Variability in these pre-analytical steps currently hampers reproducibility and complicates comparisons across studies. Establishing validated protocols and normalization strategies will be essential for accurate cross-cohort biomarker evaluation and meta-analytical integration. The miniaturization and portability of diagnostic technologies offer promising solutions for POC deployment. Advances in microfluidics, lab-on-a-chip platforms, and portable biosensors allow real-time, multiplexed detection of salivary analytes with minimal sample volume and without the need for centralized laboratory infrastructure. These tools are particularly beneficial for low-resource and remote settings and hold the potential for at-home oral cancer screening [7,14]. Future iterations of these platforms may incorporate onboard data analytics and AI-based pattern recognition to interpret pathway-related biomarker signatures. In addition, longitudinal salivary monitoring in high-risk populations, such as those with OPMDs or heavy tobacco and alcohol use, could enable dynamic risk stratification. Temporal profiling of biomarkers related to EGFR/PI3K/Akt or TGF-β/SMAD signaling could identify early molecular shifts preceding clinical manifestation, allowing for proactive surveillance and therapeutic intervention. Another critical area is the exploration of salivary exosomes and the molecular cargo they carry, such as oncogenic microRNAs, phosphorylated proteins, and DNA fragments. These exosomes function not only as biomarkers but also as active mediators of tumor progression. Through intercellular communication, salivary exosomes may facilitate processes such as immune evasion, angiogenesis, and metastasis. Their molecular composition often mirrors the activation status of signaling pathways within the tumor microenvironment, providing valuable insight into disease mechanisms and offering potential diagnostic and therapeutic targets [33]. Moving forward, the integration of salivary diagnostics with pathway-specific functional validation will be imperative.

Techniques such as CRISPR-based screening, phosphoproteomics, and pathway-reporter assays can help elucidate whether salivary alterations causally reflect intracellular signaling dysregulation or represent downstream byproducts. Functional validation is especially relevant for candidate biomarkers intended for prognostication or treatment monitoring. Finally, future success will depend on large-scale, multicenter validation studies incorporating diverse populations, robust bioinformatics pipelines, and regulatory alignment. Clinical-grade salivary diagnostics should be co-developed with clinicians, molecular biologists, and regulatory agencies to ensure safety, efficacy, and scalability. International collaboration will accelerate the transition of saliva-based tools from research to real-world clinical implementation, ultimately transforming oral cancer detection and management.

### 6.2 Potential of Salivary Biomarkers in Predicting Therapeutic Outcomes and Prognosis

While salivary biomarkers have been extensively investigated for their diagnostic utility in OSCC, their potential in predicting therapeutic outcomes and long-term prognosis is increasingly being recognized. This growing interest is supported by molecular studies highlighting how salivary analytes reflect tumor biology and dynamic treatment responses. For example, Balakittnen et al. identified a panel of salivary microRNAs that not only differentiated OSCC patients from healthy individuals but also correlated with oncogenic signaling pathways such as PI3K/Akt and TGF-β/SMAD. These findings suggest their value in evaluating disease aggressiveness and predicting treatment responses [11]. Nonaka and Wong reviewed the molecular features of cancer-derived exosomes in saliva and emphasized their potential prognostic and therapeutic roles. Their analysis revealed that salivary exosomes carry tumor-specific components, including proteins and nucleic acids, which are relevant for longitudinal monitoring in OSCC management [33]. Kok and colleagues further demonstrated that salivary exosomal cargo, such as oncogenic microRNAs and tumor-derived DNA fragments, can be effectively detected using non-invasive methods. These molecular features reflect tumor burden and minimal residual disease status and could enable real-time evaluation of treatment effectiveness and early detection of recurrence, supporting their integration into precision oncology frameworks [18]. Similarly, Zlotogorski-Hurvitz et al. provided evidence of distinct morphological and molecular signatures in salivary exosomes from OSCC patients compared to healthy controls [48]. Salfer and colleagues explored the utility of salivary cell-free DNA and found that pre-analytical factors, such as collection timing and sample processing, significantly affect cell-free DNA (cfDNA) integrity. This insight is crucial for its application in monitoring minimal residual disease following cancer therapy [38]. In addition, preliminary findings suggest that specific exosomal microRNAs such as miR-1307-5p may serve as predictive indicators of poor prognosis. A study reported that elevated salivary levels of miR-1307-5p were associated with worse clinical outcomes, suggesting its potential for early risk stratification in OSCC [56]. Panneerselvam et al. reviewed advances in salivary metabolomics and noted that certain metabolic signatures may correspond with treatment responsiveness or risk of recurrence. Although still in the exploratory stage, metabolomics may complement existing molecular biomarkers and broaden the clinical utility of salivary diagnostics [13].

Despite these promising developments, several challenges remain. Many studies are limited by small cohorts, cross-sectional designs, and lack of multicenter validation. In addition, inconsistent sampling procedures, inadequate mechanistic data, and non-standardized detection platforms hinder reproducibility and clinical translation. Therefore, future research should focus on prospective, large-scale, and multicenter investigations, as well as mechanistic studies to better understand how salivary biomarkers interact with tumor signaling and treatment pathways. By overcoming these limitations, salivary diagnostics may evolve from supplementary tools into integral components of personalized OSCC care. Their integration into clinical practice could enable early intervention, adaptive treatment strategies, and real-time disease monitoring, ultimately improving the precision and effectiveness of cancer management.

## 7 Conclusion

Salivary biomarkers present a valuable opportunity to improve the early detection and clinical management of OSCC. Traditional diagnostic methods, including biopsies and imaging, often rely on clinical expertise and may cause patient discomfort. In contrast, saliva is easily accessible, well-tolerated, and reflects both local and systemic disease-related changes, making it an ideal biofluid for non-invasive cancer diagnostics. Accumulating evidence has demonstrated that specific salivary components such as cytokines, microRNAs, exosomes, proteins, and metabolites are associated with key molecular alterations in OSCC. These biomarkers are involved in crucial biological processes, including inflammation, cell survival, immune response, and tumor progression, largely through their participation in signaling pathways such as NF-κB, MAPK, PI3K/Akt, JAK/STAT, and TGF-β/SMAD. The development of molecular technologies has enabled sensitive and specific detection of these biomarkers. Tools such as quantitative PCR, next-generation sequencing, and mass spectrometry have facilitated the identification of molecular signatures relevant to tumor biology. Moreover, the introduction of portable and automated diagnostic platforms has increased the potential for point-of-care applications, particularly in resource-limited settings or for regular screening among high-risk populations.

Despite these advancements, the clinical application of salivary biomarkers still faces several challenges. Variability in salivary composition due to personal habits, health conditions, and collection protocols can affect biomarker consistency. Many candidate biomarkers also lack validation through large-scale, multicenter studies that include diverse patient populations. In addition, mechanistic studies confirming the functional roles of these biomarkers within oncogenic pathways remain limited, which restricts their integration into clinical decision-making. To address these issues, future research should focus on standardizing pre-analytical procedures, improving normalization strategies, and establishing reproducible detection platforms. Multidisciplinary collaboration will be essential to translate laboratory findings into clinical tools. Functional studies that verify the direct involvement of salivary biomarkers in cancer-associated pathways will also be important for identifying reliable diagnostic and prognostic targets. Although diagnostic applications of salivary biomarkers in OSCC have been extensively explored, their roles in predicting therapeutic response and long-term prognosis remain underdeveloped. Emerging studies suggest that specific salivary microRNAs, exosomal cargos, and cell-free DNA may reflect treatment efficacy or disease recurrence; however, most findings are preliminary and derived from limited cohorts. There is a notable lack of prospective, multicenter, and longitudinal studies validating these biomarkers across diverse populations. Furthermore, standardized protocols, mechanistic insight, and clinical integration strategies remain insufficient. This represents a key limitation in the translational pathway of saliva-based oncology. Addressing this knowledge gap through well-designed clinical studies will be essential to fully realize the potential of salivary diagnostics not only for early detection but also for guiding therapy, monitoring response, and improving outcomes in the context of precision oral cancer care.

## Data Availability

Data sharing not applicable to this article as no datasets were generated or analyzed during the current study.
